# In This Issue

**DOI:** 10.1002/cfg.401

**Published:** 2004-04


**Comparative and Functional Genomics**
*Comp Funct Genom* 2004; **5**: 215.
Published online in Wiley InterScience (www.interscience.wiley.com). **DOI:** 10.1002/cfg.401

### In this issue

### Analysis of yeast deletion strain sensitivities to oxidative and chemical stress

Genome-wide screens of haploid *Saccharomyces cerevisiae* deletion strains exposed to sublethal doses of four chemicals (hydrogen peroxide, menadione, ibuprofen and mefloquine) were made by **Chandra Tucker** and **Stanley Fields**. They identified strains sensitive to just one chemical and strains sensitive to multiple chemicals in these screens.

### Generation of worm transposon insertion mutants

**Alexander van der Linden** and **Ronald Plasterk** present a strategy to identify and map large numbers of transposon insertions in the genome of *Caenorhabditis elegans*. Their approach makes use of the mutator strain *mut-7*, a display protocol to detect new transposon insertions, and the availability of the genome sequence of *C. elegans*.

### Antarctic genomics

Studying polar organisms can uncover not only the novel features that enable them to survive in extreme environments, but also fundamental biological principles that are common to most organisms. **Melody Clark** and colleagues review recent developments in Antarctic genomics and demonstrate the global context of such studies.

### Special Section of selected reviews from the Plant & Animal Genome XII Conference

RNAi is proving to be a valuable addition to the battery of functional genomics techniques in a number of animal species, **Louisa Matthew** discusses the progress made in applying this technique to the study of gene function in plants.

**Sean Coughlan** and colleagues discuss the different platforms currently used for global gene expression profiling, and review the first results of their comparisons of the performance of these methods in global profiling of *Arabidopsis thaliana* gene expression.

With the chicken genome sequence just completed, **Larry Cogburn** *et al.* provide a timely overview of the resources available, and the potential for future achievements, in chicken functional genomics.

**Margarita Rogatcheva** and colleagues discuss the potential for using recombineering to produce porcine models of human disease, and describe the progress they have made towards this goal.

**Marie-Michèle Cordonnier-Pratt** and co-workers describe the MAGIC database and interfaces, designed for gene discovery using ESTs and expanded for use with microarray analysis, which are being used by research programmes focusing on a wide range of organisms.

**Christopher Love** *et al.* present a series of bioinformatics tools and pipelines for processing and structuring genomic data, which have been designed for the *Brassica* research community.

Three possible models have been proposed for the origin of maize as a tetraploid. **Zuzana Swigonova** and colleagues review these theories and discuss the implications of their recent research, which supports one model in particular.

**Sandra Knapp** and co-workers argue that genomics research can benefit greatly from better integration with taxonomy (description, identification and phylogeny) and describe their efforts to unite these fields in the study of Solanaceae.

### Conference calendar

A listing of genomics-related conferences planned for September–November 2004.

Copyright © 2004 John Wiley & Sons, Ltd.

